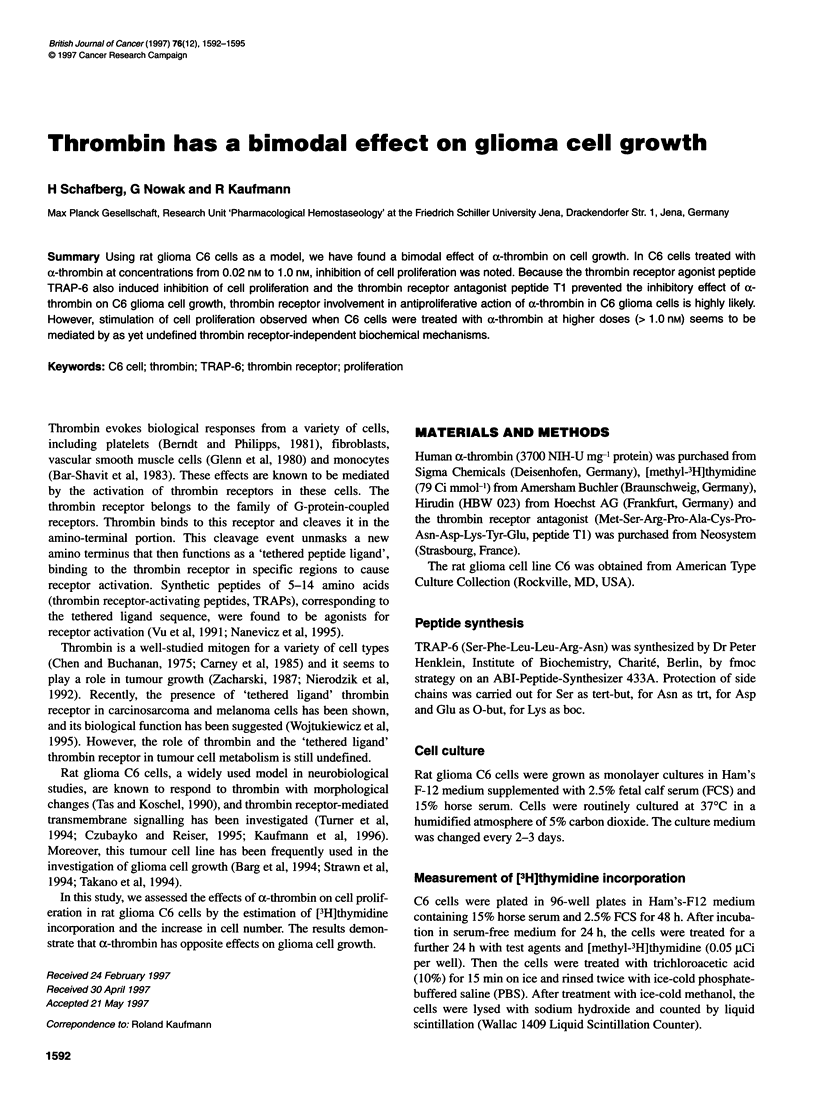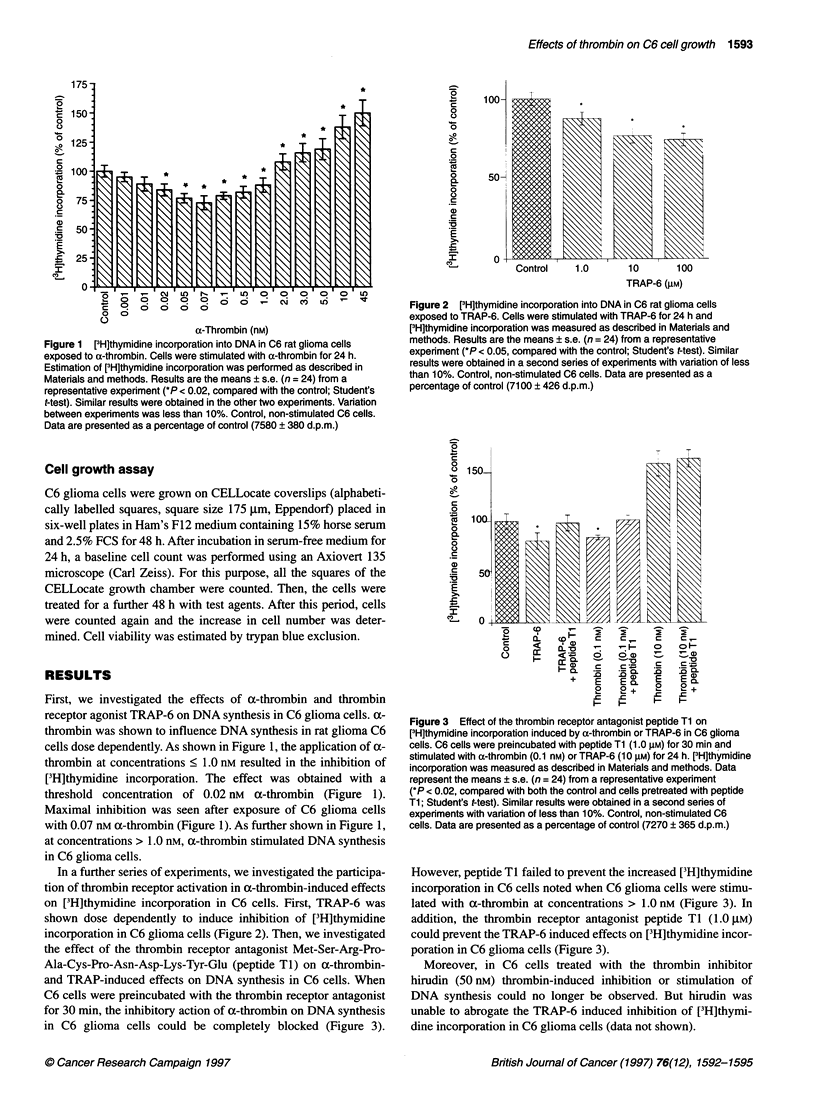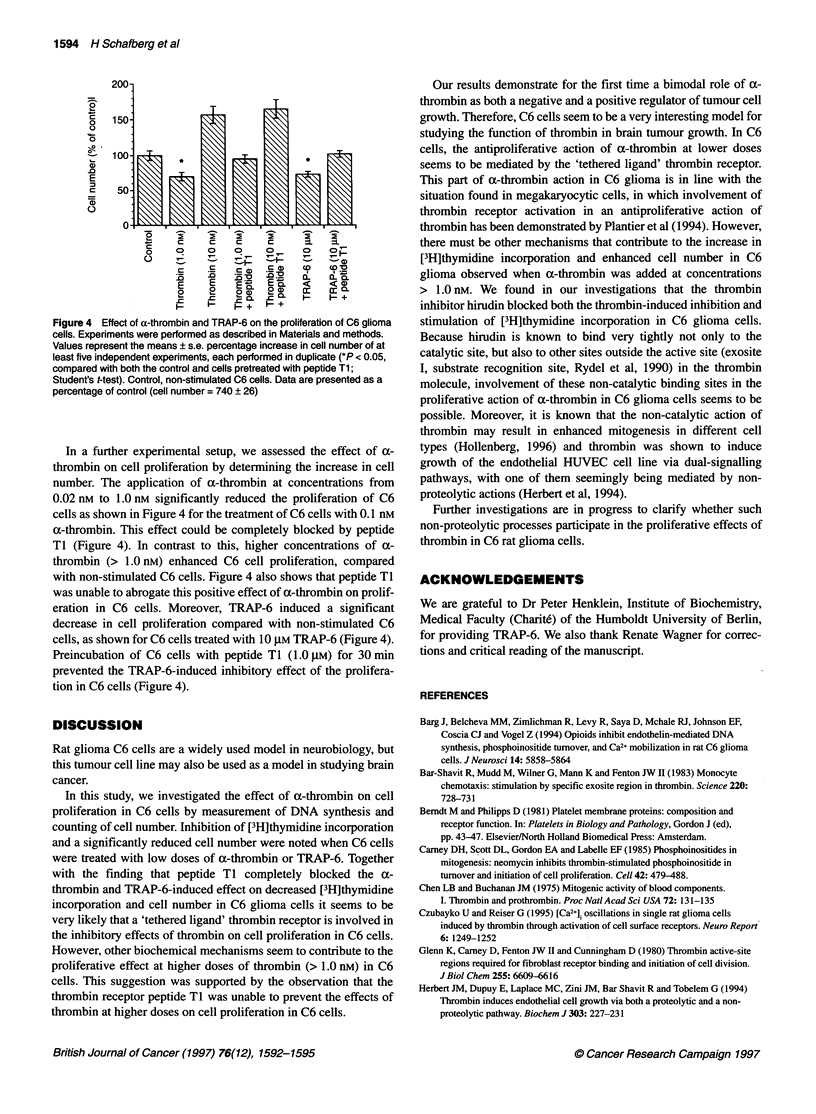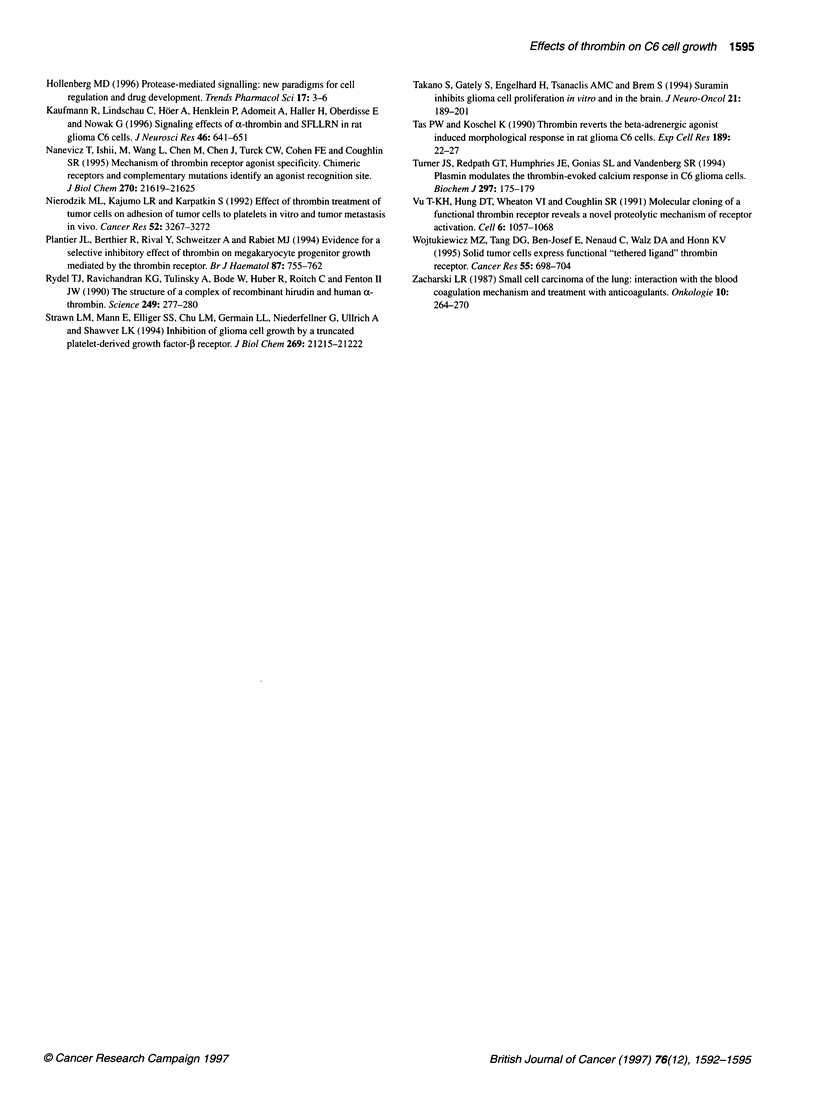# Thrombin has a bimodal effect on glioma cell growth.

**DOI:** 10.1038/bjc.1997.602

**Published:** 1997

**Authors:** H. Schafberg, G. Nowak, R. Kaufmann

**Affiliations:** Max Planck Gesellschaft, Research Unit Pharmacological Hemostaseology at the Friedrich Schiller University Jena, Germany.

## Abstract

Using rat glioma C6 cells as a model, we have found a bimodal effect of alpha-thrombin on cell growth. In C6 cells treated with alpha-thrombin at concentrations from 0.02 nM to 1.0 nM, inhibition of cell proliferation was noted. Because the thrombin receptor agonist peptide TRAP-6 also induced inhibition of cell proliferation and the thrombin receptor antagonist peptide T1 prevented the inhibitory effect of alpha-thrombin on C6 glioma cell growth, thrombin receptor involvement in antiproliferative action of alpha-thrombin in C6 glioma cells is highly likely. However, stimulation of cell proliferation observed when C6 cells were treated with alpha-thrombin at higher doses (> 1.0 nM) seems to be mediated by as yet undefined thrombin receptor-independent biochemical mechanisms.


British Journal of Cancer (1997) 76(12), 1592–1595
© 1997 Cancer Research Campaign

### Thrombin has a bimodal effect on glioma cell growth

**H Schafberg, G Nowak and R Kaufmann**

Max Planck Gesellschaft, Research Unit 'Pharmacological Hemostaseology' at the Friedrich Schiller University Jena, Drackendorfer Str. 1, Jena, Germany

**Summary** Using rat glioma C6 cells as a model, we have found a bimodal effect of α-thrombin on cell growth. In C6 cells treated with α-thrombin at concentrations from 0.02 nM to 1.0 nM, inhibition of cell proliferation was noted. Because the thrombin receptor agonist peptide TRAP-6 also induced inhibition of cell proliferation and the thrombin receptor antagonist peptide T1 prevented the inhibitory effect of α-thrombin on C6 glioma cell growth, thrombin receptor involvement in antiproliferative action of α-thrombin in C6 glioma cells is highly likely. However, stimulation of cell proliferation observed when C6 cells were treated with α-thrombin at higher doses (> 1.0 nM) seems to be mediated by as yet undefined thrombin receptor-independent biochemical mechanisms.

**Keywords:** C6 cell; thrombin; TRAP-6; thrombin receptor; proliferation

Thrombin evokes biological responses from a variety of cells, including platelets (Berndt and Philipps, 1981), fibroblasts, vascular smooth muscle cells (Glenn et al, 1980) and monocytes (Bar-Shavit et al, 1983). These effects are known to be mediated by the activation of thrombin receptors in these cells. The thrombin receptor belongs to the family of G-protein-coupled receptors. Thrombin binds to this receptor and cleaves it in the amino-terminal portion. This cleavage event unmasks a new amino terminus that then functions as a 'tethered peptide ligand', binding to the thrombin receptor in specific regions to cause receptor activation. Synthetic peptides of 5–14 amino acids (thrombin receptor-activating peptides, TRAPs), corresponding to the tethered ligand sequence, were found to be agonists for receptor activation (Vu et al, 1991; Nanevicz et al, 1995).

Thrombin is a well-studied mitogen for a variety of cell types (Chen and Buchanan, 1975; Carney et al, 1985) and it seems to play a role in tumour growth (Zacharski, 1987; Nierodzik et al, 1992). Recently, the presence of 'tethered ligand' thrombin receptor in carcinosarcoma and melanoma cells has been shown, and its biological function has been suggested (Wojtukiewicz et al, 1995). However, the role of thrombin and the 'tethered ligand' thrombin receptor in tumour cell metabolism is still undefined.

Rat glioma C6 cells, a widely used model in neurobiological studies, are known to respond to thrombin with morphological changes (Tas and Koschel, 1990), and thrombin receptor-mediated transmembrane signalling has been investigated (Turner et al, 1994; Czubayko and Reiser, 1995; Kaufmann et al, 1996). Moreover, this tumour cell line has been frequently used in the investigation of glioma cell growth (Barg et al, 1994; Strawn et al, 1994; Takano et al, 1994).

In this study, we assessed the effects of α-thrombin on cell proliferation in rat glioma C6 cells by the estimation of [$^3$H]thymidine incorporation and the increase in cell number. The results demonstrate that α-thrombin has opposite effects on glioma cell growth.

*Received 24 February 1997*
*Received 30 April 1997*
*Accepted 21 May 1997*

*Correpondence to:* Roland Kaufmann

### MATERIALS AND METHODS

Human α-thrombin (3700 NIH-U mg$^{-1}$ protein) was purchased from Sigma Chemicals (Deisenhofen, Germany), [methyl-$^3$H]thymidine (79 Ci mmol$^{-1}$) from Amersham Buchler (Braunschweig, Germany), Hirudin (HBW 023) from Hoechst AG (Frankfurt, Germany) and the thrombin receptor antagonist (Met-Ser-Arg-Pro-Ala-Cys-Pro-Asn-Asp-Lys-Tyr-Glu, peptide T1) was purchased from Neosystem (Strasbourg, France).

The rat glioma cell line C6 was obtained from American Type Culture Collection (Rockville, MD, USA).

### Peptide synthesis

TRAP-6 (Ser-Phe-Leu-Leu-Arg-Asn) was synthesized by Dr Peter Henklein, Institute of Biochemistry, Charité, Berlin, by fmoc strategy on an ABI-Peptide-Synthesizer 433A. Protection of side chains was carried out for Ser as tert-but, for Asn as trt, for Asp and Glu as O-but, for Lys as boc.

### Cell culture

Rat glioma C6 cells were grown as monolayer cultures in Ham's F-12 medium supplemented with 2.5% fetal calf serum (FCS) and 15% horse serum. Cells were routinely cultured at 37°C in a humidified atmosphere of 5% carbon dioxide. The culture medium was changed every 2–3 days.

### Measurement of [$^3$H]thymidine incorporation

C6 cells were plated in 96-well plates in Ham's-F12 medium containing 15% horse serum and 2.5% FCS for 48 h. After incubation in serum-free medium for 24 h, the cells were treated for a further 24 h with test agents and [methyl-$^3$H]thymidine (0.05 µCi per well). Then the cells were treated with trichloroacetic acid (10%) for 15 min on ice and rinsed twice with ice-cold phosphate-buffered saline (PBS). After treatment with ice-cold methanol, the cells were lysed with sodium hydroxide and counted by liquid scintillation (Wallac 1409 Liquid Scintillation Counter).

**Figure 1** [$^3$H]thymidine incorporation into DNA in C6 rat glioma cells exposed to α-thrombin. Cells were stimulated with α-thrombin for 24 h. Estimation of [$^3$H]thymidine incorporation was performed as described in Materials and methods. Results are the means ± s.e. ($n$ = 24) from a representative experiment (*$P$ < 0.02, compared with the control; Student's $t$-test). Similar results were obtained in the other two experiments. Variation between experiments was less than 10%. Control, non-stimulated C6 cells. Data are presented as a percentage of control (7580 ± 380 d.p.m.)

**Figure 2** [$^3$H]thymidine incorporation into DNA in C6 rat glioma cells exposed to TRAP-6. Cells were stimulated with TRAP-6 for 24 h and [$^3$H]thymidine incorporation was measured as described in Materials and methods. Results are the means ± s.e. ($n$ = 24) from a representative experiment (*$P$ < 0.05, compared with the control; Student's $t$-test). Similar results were obtained in a second series of experiments with variation of less than 10%. Control, non-stimulated C6 cells. Data are presented as a percentage of control (7100 ± 426 d.p.m.)

**Figure 3** Effect of the thrombin receptor antagonist peptide T1 on [$^3$H]thymidine incorporation induced by α-thrombin or TRAP-6 in C6 glioma cells. C6 cells were preincubated with peptide T1 (1.0 μM) for 30 min and stimulated with α-thrombin (0.1 nM) or TRAP-6 (10 μM) for 24 h. [$^3$H]thymidine incorporation was measured as described in Materials and methods. Data represent the means ± s.e. ($n$ = 24) from a representative experiment (*$P$ < 0.02, compared with both the control and cells pretreated with peptide T1; Student's $t$-test). Similar results were obtained in a second series of experiments with variation of less than 10%. Control, non-stimulated C6 cells. Data are presented as a percentage of control (7270 ± 365 d.p.m.)

### Cell growth assay

C6 glioma cells were grown on CELLocate coverslips (alphabetically labelled squares, square size 175 μm, Eppendorf) placed in six-well plates in Ham's F12 medium containing 15% horse serum and 2.5% FCS for 48 h. After incubation in serum-free medium for 24 h, a baseline cell count was performed using an Axiovert 135 microscope (Carl Zeiss). For this purpose, all the squares of the CELLocate growth chamber were counted. Then, the cells were treated for a further 48 h with test agents. After this period, cells were counted again and the increase in cell number was determined. Cell viability was estimated by trypan blue exclusion.

### RESULTS

First, we investigated the effects of α-thrombin and thrombin receptor agonist TRAP-6 on DNA synthesis in C6 glioma cells. α-thrombin was shown to influence DNA synthesis in rat glioma C6 cells dose dependently. As shown in Figure 1, the application of α-thrombin at concentrations ≤ 1.0 nM resulted in the inhibition of [$^3$H]thymidine incorporation. The effect was obtained with a threshold concentration of 0.02 nM α-thrombin (Figure 1). Maximal inhibition was seen after exposure of C6 glioma cells with 0.07 nM α-thrombin (Figure 1). As further shown in Figure 1, at concentrations > 1.0 nM, α-thrombin stimulated DNA synthesis in C6 glioma cells.

In a further series of experiments, we investigated the participation of thrombin receptor activation in α-thrombin-induced effects on [$^3$H]thymidine incorporation in C6 cells. First, TRAP-6 was shown dose dependently to induce inhibition of [$^3$H]thymidine incorporation in C6 glioma cells (Figure 2). Then, we investigated the effect of the thrombin receptor antagonist Met-Ser-Arg-Pro-Ala-Cys-Pro-Asn-Asp-Lys-Tyr-Glu (peptide T1) on α-thrombin- and TRAP-induced effects on DNA synthesis in C6 cells. When C6 cells were preincubated with the thrombin receptor antagonist for 30 min, the inhibitory action of α-thrombin on DNA synthesis in C6 glioma cells could be completely blocked (Figure 3). However, peptide T1 failed to prevent the increased [$^3$H]thymidine incorporation in C6 cells noted when C6 glioma cells were stimulated with α-thrombin at concentrations > 1.0 nM (Figure 3). In addition, the thrombin receptor antagonist peptide T1 (1.0 μM) could prevent the TRAP-6 induced effects on [$^3$H]thymidine incorporation in C6 glioma cells (Figure 3).

Moreover, in C6 cells treated with the thrombin inhibitor hirudin (50 nM) thrombin-induced inhibition or stimulation of DNA synthesis could no longer be observed. But hirudin was unable to abrogate the TRAP-6 induced inhibition of [$^3$H]thymidine incorporation in C6 glioma cells (data not shown).

**Figure 4** Effect of α-thrombin and TRAP-6 on the proliferation of C6 glioma cells. Experiments were performed as described in Materials and methods. Values represent the means ± s.e. percentage increase in cell number of at least five independent experiments, each performed in duplicate (*$P < 0.05$, compared with both the control and cells pretreated with peptide T1; Student's *t*-test). Control, non-stimulated C6 cells. Data are presented as a percentage of control (cell number = 740 ± 26)

In a further experimental setup, we assessed the effect of α-thrombin on cell proliferation by determining the increase in cell number. The application of α-thrombin at concentrations from 0.02 nM to 1.0 nM significantly reduced the proliferation of C6 cells as shown in Figure 4 for the treatment of C6 cells with 0.1 nM α-thrombin. This effect could be completely blocked by peptide T1 (Figure 4). In contrast to this, higher concentrations of α-thrombin (> 1.0 nM) enhanced C6 cell proliferation, compared with non-stimulated C6 cells. Figure 4 also shows that peptide T1 was unable to abrogate this positive effect of α-thrombin on proliferation in C6 cells. Moreover, TRAP-6 induced a significant decrease in cell proliferation compared with non-stimulated C6 cells, as shown for C6 cells treated with 10 μM TRAP-6 (Figure 4). Preincubation of C6 cells with peptide T1 (1.0 μM) for 30 min prevented the TRAP-6-induced inhibitory effect of the proliferation in C6 cells (Figure 4).

### DISCUSSION

Rat glioma C6 cells are a widely used model in neurobiology, but this tumour cell line may also be used as a model in studying brain cancer.

In this study, we investigated the effect of α-thrombin on cell proliferation in C6 cells by measurement of DNA synthesis and counting of cell number. Inhibition of [$^3$H]thymidine incorporation and a significantly reduced cell number were noted when C6 cells were treated with low doses of α-thrombin or TRAP-6. Together with the finding that peptide T1 completely blocked the α-thrombin and TRAP-6-induced effect on decreased [$^3$H]thymidine incorporation and cell number in C6 glioma cells it seems to be very likely that a 'tethered ligand' thrombin receptor is involved in the inhibitory effects of thrombin on cell proliferation in C6 cells. However, other biochemical mechanisms seem to contribute to the proliferative effect at higher doses of thrombin (> 1.0 nM) in C6 cells. This suggestion was supported by the observation that the thrombin receptor peptide T1 was unable to prevent the effects of thrombin at higher doses on cell proliferation in C6 cells.

Our results demonstrate for the first time a bimodal role of α-thrombin as both a negative and a positive regulator of tumour cell growth. Therefore, C6 cells seem to be a very interesting model for studying the function of thrombin in brain tumour growth. In C6 cells, the antiproliferative action of α-thrombin at lower doses seems to be mediated by the 'tethered ligand' thrombin receptor. This part of α-thrombin action in C6 glioma is in line with the situation found in megakaryocytic cells, in which involvement of thrombin receptor activation in an antiproliferative action of thrombin has been demonstrated by Plantier et al (1994). However, there must be other mechanisms that contribute to the increase in [$^3$H]thymidine incorporation and enhanced cell number in C6 glioma observed when α-thrombin was added at concentrations > 1.0 nM. We found in our investigations that the thrombin inhibitor hirudin blocked both the thrombin-induced inhibition and stimulation of [$^3$H]thymidine incorporation in C6 glioma cells. Because hirudin is known to bind very tightly not only to the catalytic site, but also to other sites outside the active site (exosite I, substrate recognition site, Rydel et al, 1990) in the thrombin molecule, involvement of these non-catalytic binding sites in the proliferative action of α-thrombin in C6 glioma cells seems to be possible. Moreover, it is known that the non-catalytic action of thrombin may result in enhanced mitogenesis in different cell types (Hollenberg, 1996) and thrombin was shown to induce growth of the endothelial HUVEC cell line via dual-signalling pathways, with one of them seemingly being mediated by non-proteolytic actions (Herbert et al, 1994).

Further investigations are in progress to clarify whether such non-proteolytic processes participate in the proliferative effects of thrombin in C6 rat glioma cells.

### ACKNOWLEDGEMENTS

We are grateful to Dr Peter Henklein, Institute of Biochemistry, Medical Faculty (Charité) of the Humboldt University of Berlin, for providing TRAP-6. We also thank Renate Wagner for corrections and critical reading of the manuscript.

### REFERENCES

Barg J, Belcheva MM, Zimlichman R, Levy R, Saya D, Mchale RJ, Johnson EF, Coscia CJ and Vogel Z (1994) Opioids inhibit endothelin-mediated DNA synthesis, phosphoinositide turnover, and $Ca^{2+}$ mobilization in rat C6 glioma cells. *J Neurosci* **14**: 5858–5864

Bar-Shavit R, Mudd M, Wilner G, Mann K and Fenton JW II (1983) Monocyte chemotaxis: stimulation by specific exosite region in thrombin. *Science* **220**: 728–731

Berndt M and Philipps D (1981) Platelet membrane proteins: composition and receptor function. In: *Platelets in Biology and Pathology*, Gordon J (ed), pp. 43–47. Elsevier/North Holland Biomedical Press: Amsterdam.

Carney DH, Scott DL, Gordon EA and Labelle EF (1985) Phosphoinositides in mitogenesis: neomycin inhibits thrombin-stimulated phosphoinositide in turnover and initiation of cell proliferation. *Cell* **42**: 479–488.

Chen LB and Buchanan JM (1975) Mitogenic activity of blood components. I. Thrombin and prothrombin. *Proc Natl Acad Sci USA* **72**: 131–135

Czubayko U and Reiser G (1995) $[Ca^{2+}]_i$ oscillations in single rat glioma cells induced by thrombin through activation of cell surface receptors. *Neuro Report* **6**: 1249–1252

Glenn K, Carney D, Fenton JW II and Cunningham D (1980) Thrombin active-site regions required for fibroblast receptor binding and initiation of cell division. *J Biol Chem* **255**: 6609–6616

Herbert JM, Dupuy E, Laplace MC, Zini JM, Bar Shavit R and Tobelem G (1994) Thrombin induces endothelial cell growth via both a proteolytic and a non-proteolytic pathway. *Biochem J* **303**: 227–231

© Cancer Research Campaign 1997

Hollenberg MD (1996) Protease-mediated signalling: new paradigms for cell regulation and drug development. *Trends Pharmacol Sci* **17:** 3–6

Kaufmann R, Lindschau C, Höer A, Henklein P, Adomeit A, Haller H, Oberdisse E and Nowak G (1996) Signaling effects of α-thrombin and SFLLRN in rat glioma C6 cells. *J Neurosci Res* **46:** 641–651

Nanevicz T, Ishii, M, Wang L, Chen M, Chen J, Turck CW, Cohen FE and Coughlin SR (1995) Mechanism of thrombin receptor agonist specificity. Chimeric receptors and complementary mutations identify an agonist recognition site. *J Biol Chem* **270:** 21619–21625

Nierodzik ML, Kajumo LR and Karpatkin S (1992) Effect of thrombin treatment of tumor cells on adhesion of tumor cells to platelets in vitro and tumor metastasis in vivo. *Cancer Res* **52:** 3267–3272

Plantier JL, Berthier R, Rival Y, Schweitzer A and Rabiet MJ (1994) Evidence for a selective inhibitory effect of thrombin on megakaryocyte progenitor growth mediated by the thrombin receptor. *Br J Haematol* **87:** 755–762

Rydel TJ, Ravichandran KG, Tulinsky A, Bode W, Huber R, Roitch C and Fenton II JW (1990) The structure of a complex of recombinant hirudin and human α-thrombin. *Science* **249:** 277–280

Strawn LM, Mann E, Elliger SS, Chu LM, Germain LL, Niederfellner G, Ullrich A and Shawver LK (1994) Inhibition of glioma cell growth by a truncated platelet-derived growth factor-β receptor. *J Biol Chem* **269:** 21215–21222

Takano S, Gately S, Engelhard H, Tsanaclis AMC and Brem S (1994) Suramin inhibits glioma cell proliferation *in vitro* and in the brain. *J Neuro-Oncol* **21:** 189–201

Tas PW and Koschel K (1990) Thrombin reverts the beta-adrenergic agonist induced morphological response in rat glioma C6 cells. *Exp Cell Res* **189:** 22–27

Turner JS, Redpath GT, Humphries JE, Gonias SL and Vandenberg SR (1994) Plasmin modulates the thrombin-evoked calcium response in C6 glioma cells. *Biochem J* **297:** 175–179

Vu T-KH, Hung DT, Wheaton VI and Coughlin SR (1991) Molecular cloning of a functional thrombin receptor reveals a novel proteolytic mechanism of receptor activation. *Cell* **6:** 1057–1068

Wojtukiewicz MZ, Tang DG, Ben-Josef E, Nenaud C, Walz DA and Honn KV (1995) Solid tumor cells express functional "tethered ligand" thrombin receptor. *Cancer Res* **55:** 698–704

Zacharski LR (1987) Small cell carcinoma of the lung: interaction with the blood coagulation mechanism and treatment with anticoagulants. *Onkologie* **10:** 264–270

© Cancer Research Campaign 1997